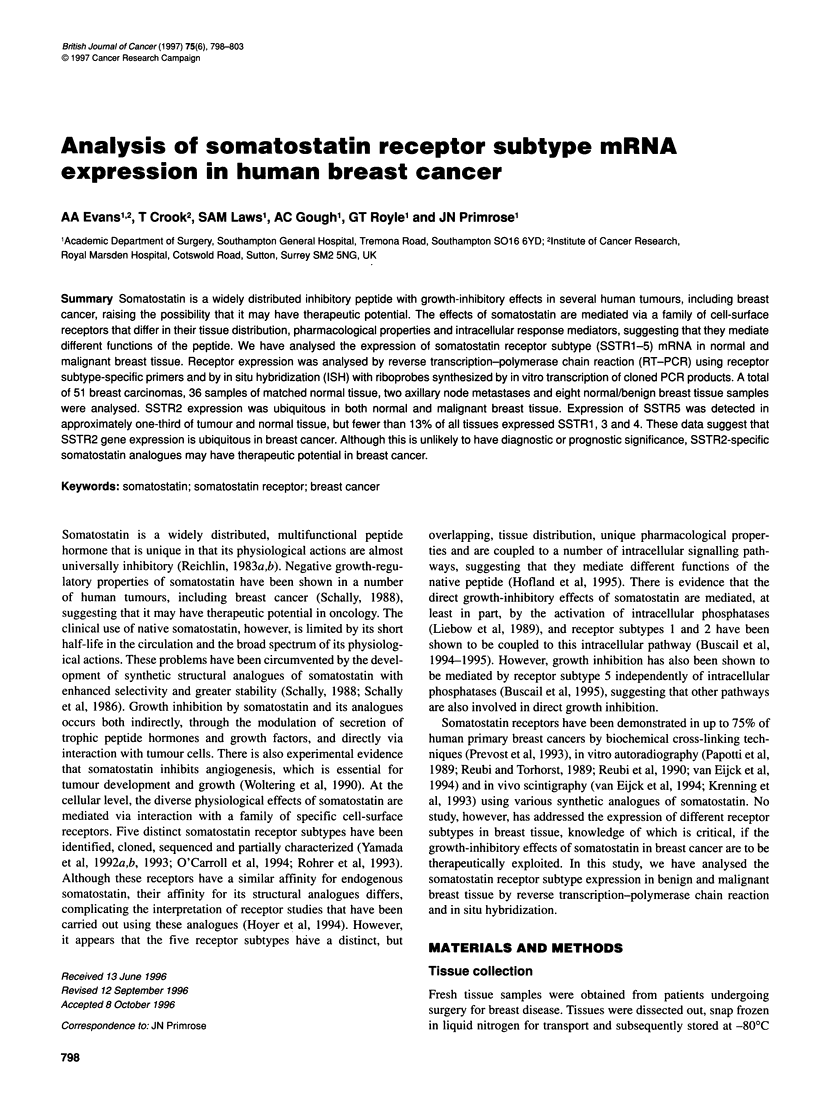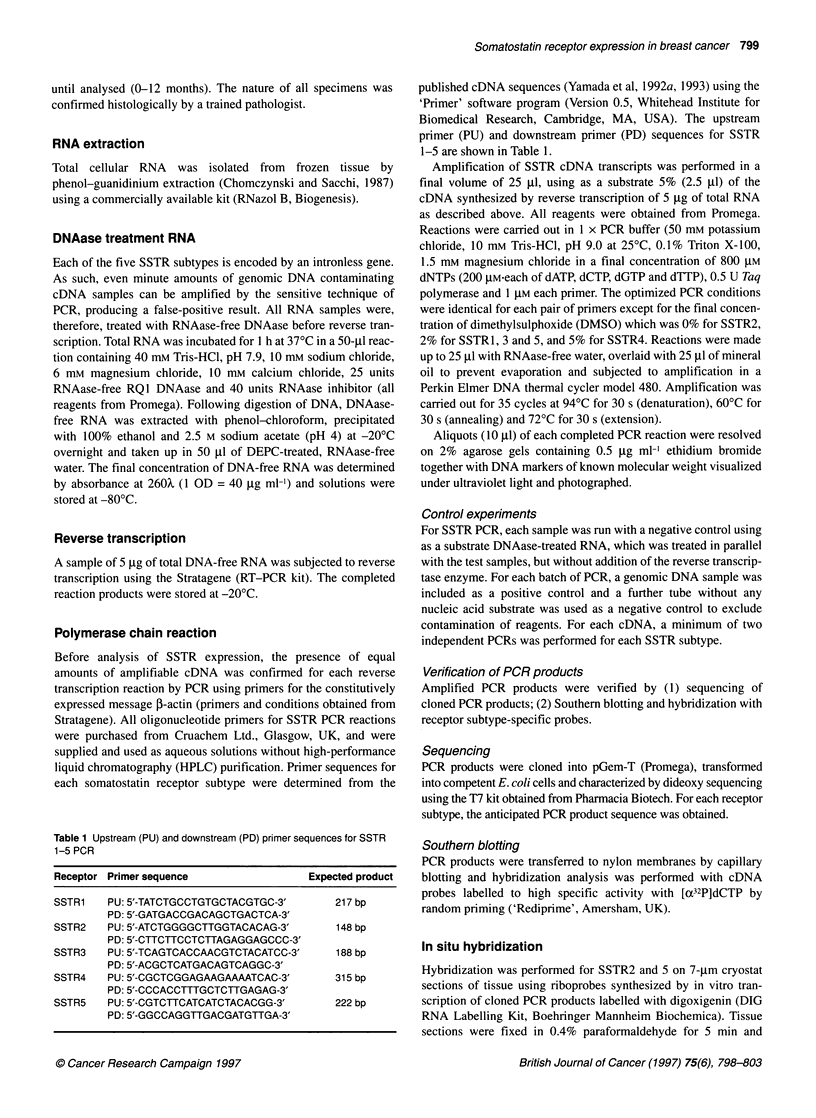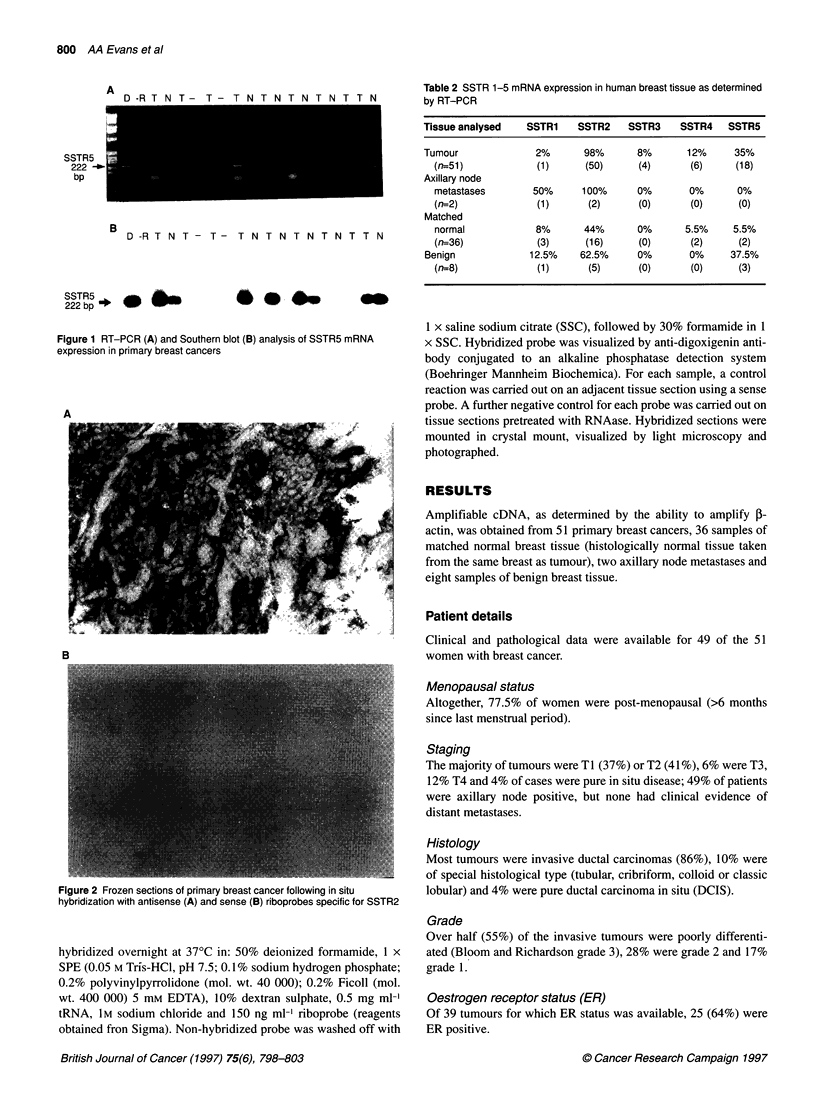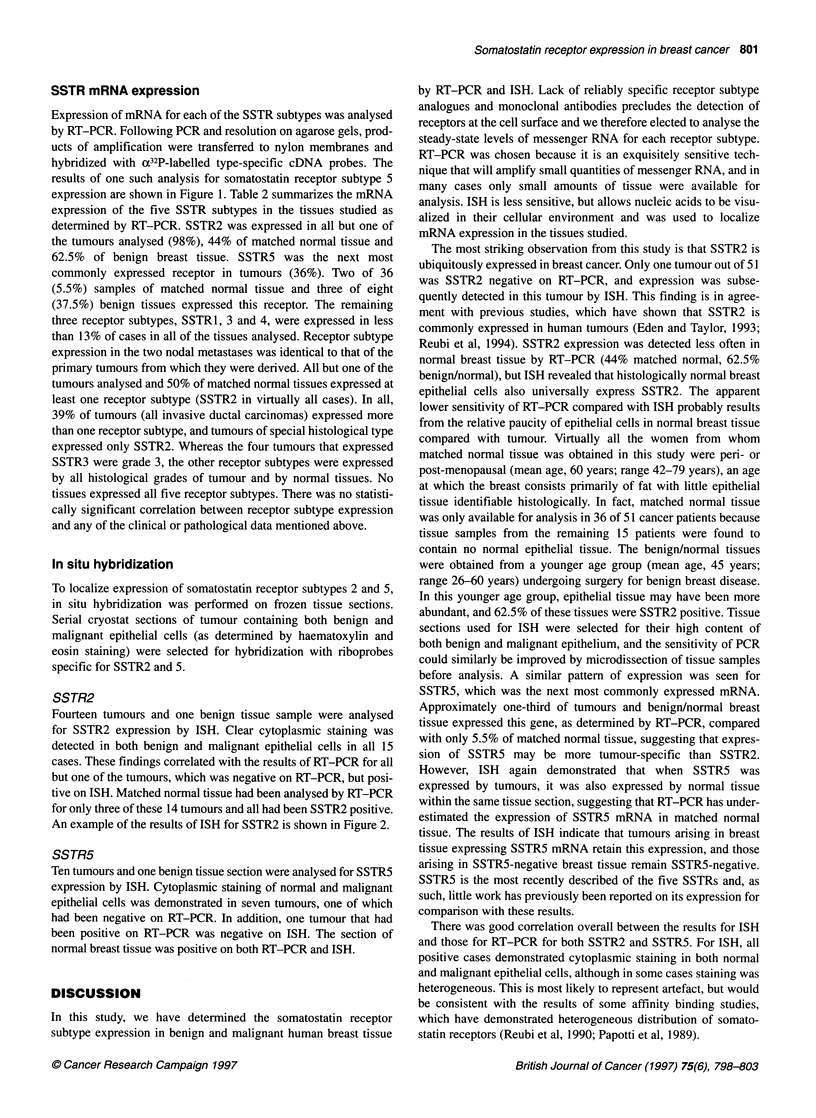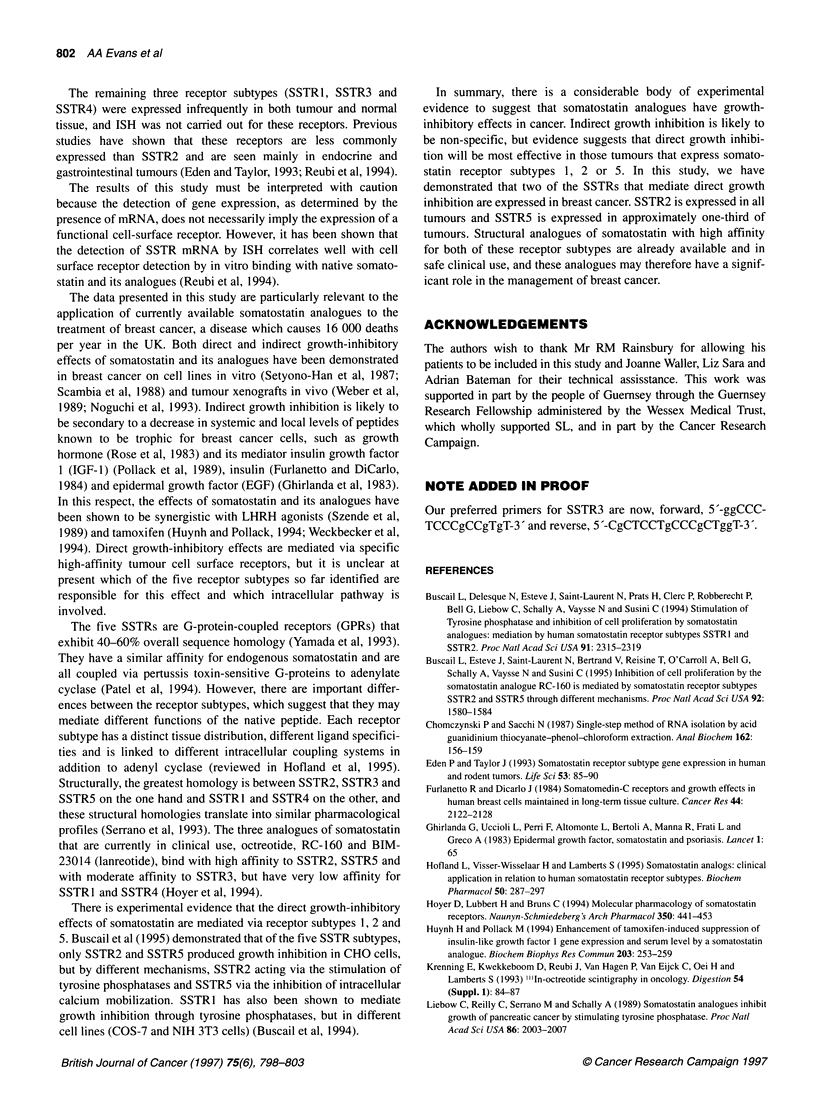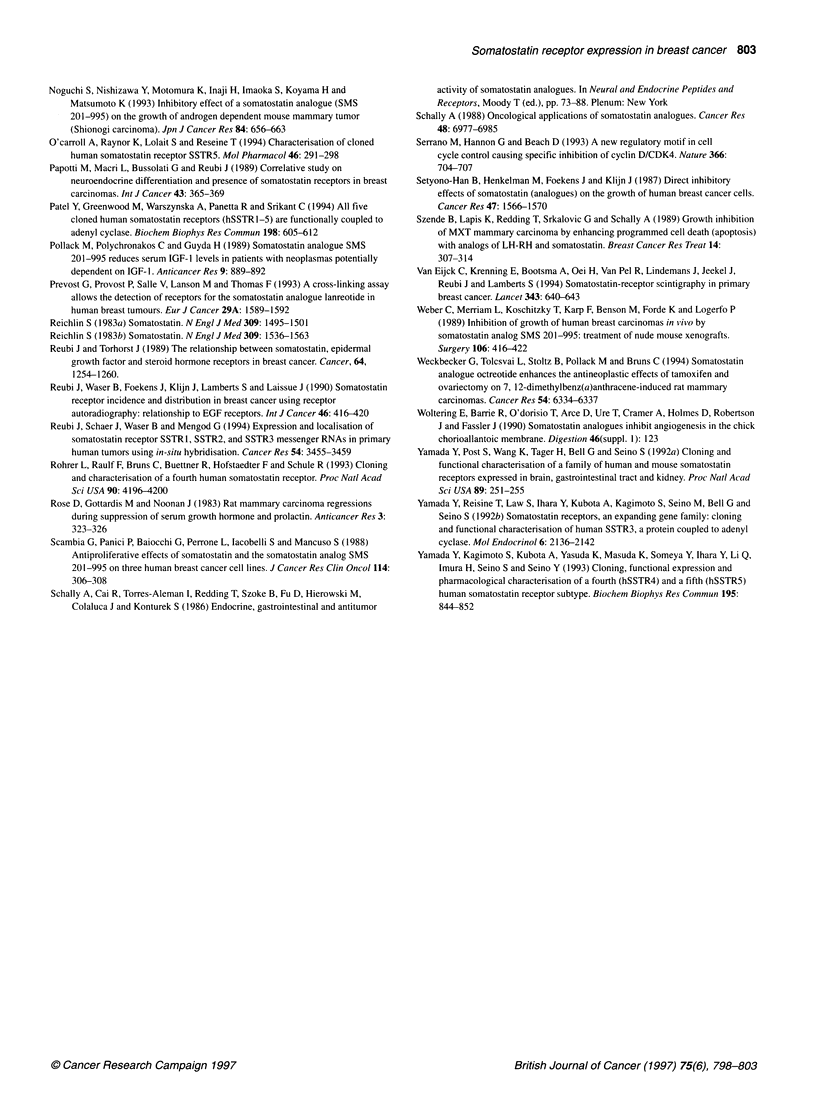# Analysis of somatostatin receptor subtype mRNA expression in human breast cancer.

**DOI:** 10.1038/bjc.1997.143

**Published:** 1997

**Authors:** A. A. Evans, T. Crook, S. A. Laws, A. C. Gough, G. T. Royle, J. N. Primrose

**Affiliations:** Academic Department of Surgery, Southampton General Hospital, UK.

## Abstract

**Images:**


British Journal of Cancer (1997) 75(6), 798–803
© 1997 Cancer Research Campaign

### Analysis of somatostatin receptor subtype mRNA expression in human breast cancer

**AA Evans[1,2], T Crook[2], SAM Laws[1], AC Gough[1], GT Royle[1] and JN Primrose[1]**

[1]Academic Department of Surgery, Southampton General Hospital, Tremona Road, Southampton SO16 6YD; [2]Institute of Cancer Research, Royal Marsden Hospital, Cotswold Road, Sutton, Surrey SM2 5NG, UK

**Summary** Somatostatin is a widely distributed inhibitory peptide with growth-inhibitory effects in several human tumours, including breast cancer, raising the possibility that it may have therapeutic potential. The effects of somatostatin are mediated via a family of cell-surface receptors that differ in their tissue distribution, pharmacological properties and intracellular response mediators, suggesting that they mediate different functions of the peptide. We have analysed the expression of somatostatin receptor subtype (SSTR1–5) mRNA in normal and malignant breast tissue. Receptor expression was analysed by reverse transcription–polymerase chain reaction (RT–PCR) using receptor subtype-specific primers and by in situ hybridization (ISH) with riboprobes synthesized by in vitro transcription of cloned PCR products. A total of 51 breast carcinomas, 36 samples of matched normal tissue, two axillary node metastases and eight normal/benign breast tissue samples were analysed. SSTR2 expression was ubiquitous in both normal and malignant breast tissue. Expression of SSTR5 was detected in approximately one-third of tumour and normal tissue, but fewer than 13% of all tissues expressed SSTR1, 3 and 4. These data suggest that SSTR2 gene expression is ubiquitous in breast cancer. Although this is unlikely to have diagnostic or prognostic significance, SSTR2-specific somatostatin analogues may have therapeutic potential in breast cancer.

**Keywords:** somatostatin; somatostatin receptor; breast cancer

Somatostatin is a widely distributed, multifunctional peptide hormone that is unique in that its physiological actions are almost universally inhibitory (Reichlin, 1983*a,b*). Negative growth-regulatory properties of somatostatin have been shown in a number of human tumours, including breast cancer (Schally, 1988), suggesting that it may have therapeutic potential in oncology. The clinical use of native somatostatin, however, is limited by its short half-life in the circulation and the broad spectrum of its physiological actions. These problems have been circumvented by the development of synthetic structural analogues of somatostatin with enhanced selectivity and greater stability (Schally, 1988; Schally et al, 1986). Growth inhibition by somatostatin and its analogues occurs both indirectly, through the modulation of secretion of trophic peptide hormones and growth factors, and directly via interaction with tumour cells. There is also experimental evidence that somatostatin inhibits angiogenesis, which is essential for tumour development and growth (Woltering et al, 1990). At the cellular level, the diverse physiological effects of somatostatin are mediated via interaction with a family of specific cell-surface receptors. Five distinct somatostatin receptor subtypes have been identified, cloned, sequenced and partially characterized (Yamada et al, 1992*a,b*, 1993; O'Carroll et al, 1994; Rohrer et al, 1993). Although these receptors have a similar affinity for endogenous somatostatin, their affinity for its structural analogues differs, complicating the interpretation of receptor studies that have been carried out using these analogues (Hoyer et al, 1994). However, it appears that the five receptor subtypes have a distinct, but overlapping, tissue distribution, unique pharmacological properties and are coupled to a number of intracellular signalling pathways, suggesting that they mediate different functions of the native peptide (Hofland et al, 1995). There is evidence that the direct growth-inhibitory effects of somatostatin are mediated, at least in part, by the activation of intracellular phosphatases (Liebow et al, 1989), and receptor subtypes 1 and 2 have been shown to be coupled to this intracellular pathway (Buscail et al, 1994–1995). However, growth inhibition has also been shown to be mediated by receptor subtype 5 independently of intracellular phosphatases (Buscail et al, 1995), suggesting that other pathways are also involved in direct growth inhibition.

Somatostatin receptors have been demonstrated in up to 75% of human primary breast cancers by biochemical cross-linking techniques (Prevost et al, 1993), in vitro autoradiography (Papotti et al, 1989; Reubi and Torhorst, 1989; Reubi et al, 1990; van Eijck et al, 1994) and in vivo scintigraphy (van Eijck et al, 1994; Krenning et al, 1993) using various synthetic analogues of somatostatin. No study, however, has addressed the expression of different receptor subtypes in breast tissue, knowledge of which is critical, if the growth-inhibitory effects of somatostatin in breast cancer are to be therapeutically exploited. In this study, we have analysed the somatostatin receptor subtype expression in benign and malignant breast tissue by reverse transcription–polymerase chain reaction and in situ hybridization.

### MATERIALS AND METHODS

### Tissue collection

Fresh tissue samples were obtained from patients undergoing surgery for breast disease. Tissues were dissected out, snap frozen in liquid nitrogen for transport and subsequently stored at –80°C

*Received 13 June 1996*
*Revised 12 September 1996*
*Accepted 8 October 1996*

*Correspondence to:* JN Primrose until analysed (0–12 months). The nature of all specimens was confirmed histologically by a trained pathologist.

### RNA extraction

Total cellular RNA was isolated from frozen tissue by phenol–guanidinium extraction (Chomczynski and Sacchi, 1987) using a commercially available kit (RNazol B, Biogenesis).

### DNAase treatment RNA

Each of the five SSTR subtypes is encoded by an intronless gene. As such, even minute amounts of genomic DNA contaminating cDNA samples can be amplified by the sensitive technique of PCR, producing a false-positive result. All RNA samples were, therefore, treated with RNAase-free DNAase before reverse transcription. Total RNA was incubated for 1 h at 37°C in a 50-μl reaction containing 40 mM Tris-HCl, pH 7.9, 10 mM sodium chloride, 6 mM magnesium chloride, 10 mM calcium chloride, 25 units RNAase-free RQ1 DNAase and 40 units RNAase inhibitor (all reagents from Promega). Following digestion of DNA, DNAase-free RNA was extracted with phenol–chloroform, precipitated with 100% ethanol and 2.5 M sodium acetate (pH 4) at –20°C overnight and taken up in 50 μl of DEPC-treated, RNAase-free water. The final concentration of DNA-free RNA was determined by absorbance at 260λ (1 OD = 40 μg $ml^{-1}$) and solutions were stored at –80°C.

### Reverse transcription

A sample of 5 μg of total DNA-free RNA was subjected to reverse transcription using the Stratagene (RT–PCR kit). The completed reaction products were stored at –20°C.

### Polymerase chain reaction

Before analysis of SSTR expression, the presence of equal amounts of amplifiable cDNA was confirmed for each reverse transcription reaction by PCR using primers for the constitutively expressed message β-actin (primers and conditions obtained from Stratagene). All oligonucleotide primers for SSTR PCR reactions were purchased from Cruachem Ltd., Glasgow, UK, and were supplied and used as aqueous solutions without high-performance liquid chromatography (HPLC) purification. Primer sequences for each somatostatin receptor subtype were determined from the published cDNA sequences (Yamada et al, 1992*a*, 1993) using the 'Primer' software program (Version 0.5, Whitehead Institute for Biomedical Research, Cambridge, MA, USA). The upstream primer (PU) and downstream primer (PD) sequences for SSTR 1–5 are shown in Table 1.

Amplification of SSTR cDNA transcripts was performed in a final volume of 25 μl, using as a substrate 5% (2.5 μl) of the cDNA synthesized by reverse transcription of 5 μg of total RNA as described above. All reagents were obtained from Promega. Reactions were carried out in 1 × PCR buffer (50 mM potassium chloride, 10 mM Tris-HCl, pH 9.0 at 25°C, 0.1% Triton X-100, 1.5 mM magnesium chloride in a final concentration of 800 μM dNTPs (200 μM·each of dATP, dCTP, dGTP and dTTP), 0.5 U *Taq* polymerase and 1 μM each primer. The optimized PCR conditions were identical for each pair of primers except for the final concentration of dimethylsulphoxide (DMSO) which was 0% for SSTR2, 2% for SSTR1, 3 and 5, and 5% for SSTR4. Reactions were made up to 25 μl with RNAase-free water, overlaid with 25 μl of mineral oil to prevent evaporation and subjected to amplification in a Perkin Elmer DNA thermal cycler model 480. Amplification was carried out for 35 cycles at 94°C for 30 s (denaturation), 60°C for 30 s (annealing) and 72°C for 30 s (extension).

Aliquots (10 μl) of each completed PCR reaction were resolved on 2% agarose gels containing 0.5 μg $ml^{-1}$ ethidium bromide together with DNA markers of known molecular weight visualized under ultraviolet light and photographed.

**Table 1** Upstream (PU) and downstream (PD) primer sequences for SSTR 1–5 PCR

| Receptor | Primer sequence | Expected product |
|---|---|---|
| SSTR1 | PU: 5′-TATCTGCCTGTGCTACGTGC-3′<br>PD: 5′-GATGACCGACAGCTGACTCA-3′ | 217 bp |
| SSTR2 | PU: 5′-ATCTGGGGCTTGGTACACAG-3′<br>PD: 5′-CTTCTTCCTCTTAGAGGAGCCC-3′ | 148 bp |
| SSTR3 | PU: 5′-TCAGTCACCAACGTCTACATCC-3′<br>PD: 5′-ACGCTCATGACAGTCAGGC-3′ | 188 bp |
| SSTR4 | PU: 5′-CGCTCGGAGAAGAAAATCAC-3′<br>PD: 5′-CCCACCTTTGCTCTTGAGAG-3′ | 315 bp |
| SSTR5 | PU: 5′-CGTCTTCATCATCTACACGG-3′<br>PD: 5′-GGCCAGGTTGACGATGTTGA-3′ | 222 bp |

### *Control experiments*

For SSTR PCR, each sample was run with a negative control using as a substrate DNAase-treated RNA, which was treated in parallel with the test samples, but without addition of the reverse transcriptase enzyme. For each batch of PCR, a genomic DNA sample was included as a positive control and a further tube without any nucleic acid substrate was used as a negative control to exclude contamination of reagents. For each cDNA, a minimum of two independent PCRs was performed for each SSTR subtype.

### *Verification of PCR products*

Amplified PCR products were verified by (1) sequencing of cloned PCR products; (2) Southern blotting and hybridization with receptor subtype-specific probes.

### *Sequencing*

PCR products were cloned into pGem-T (Promega), transformed into competent *E. coli* cells and characterized by dideoxy sequencing using the T7 kit obtained from Pharmacia Biotech. For each receptor subtype, the anticipated PCR product sequence was obtained.

### *Southern blotting*

PCR products were transferred to nylon membranes by capillary blotting and hybridization analysis was performed with cDNA probes labelled to high specific activity with [$\alpha^{32}$P]dCTP by random priming ('Rediprime', Amersham, UK).

### In situ hybridization

Hybridization was performed for SSTR2 and 5 on 7-μm cryostat sections of tissue using riboprobes synthesized by in vitro transcription of cloned PCR products labelled with digoxigenin (DIG RNA Labelling Kit, Boehringer Mannheim Biochemica). Tissue sections were fixed in 0.4% paraformaldehyde for 5 min and

© Cancer Research Campaign 1997

**Figure 1** RT–PCR (**A**) and Southern blot (**B**) analysis of SSTR5 mRNA expression in primary breast cancers

**Figure 2** Frozen sections of primary breast cancer following in situ hybridization with antisense (**A**) and sense (**B**) riboprobes specific for SSTR2

hybridized overnight at 37°C in: 50% deionized formamide, 1 × SPE (0.05 M Tris-HCl, pH 7.5; 0.1% sodium hydrogen phosphate; 0.2% polyvinylpyrrolidone (mol. wt. 40 000); 0.2% Ficoll (mol. wt. 400 000) 5 mM EDTA), 10% dextran sulphate, 0.5 mg $ml^{-1}$ tRNA, 1M sodium chloride and 150 ng $ml^{-1}$ riboprobe (reagents obtained fron Sigma). Non-hybridized probe was washed off with 1 × saline sodium citrate (SSC), followed by 30% formamide in 1 × SSC. Hybridized probe was visualized by anti-digoxigenin antibody conjugated to an alkaline phosphatase detection system (Boehringer Mannheim Biochemica). For each sample, a control reaction was carried out on an adjacent tissue section using a sense probe. A further negative control for each probe was carried out on tissue sections pretreated with RNAase. Hybridized sections were mounted in crystal mount, visualized by light microscopy and photographed.

**Table 2** SSTR 1–5 mRNA expression in human breast tissue as determined by RT–PCR

| Tissue analysed | SSTR1 | SSTR2 | SSTR3 | SSTR4 | SSTR5 |
|---|---|---|---|---|---|
| Tumour | 2% | 98% | 8% | 12% | 35% |
| (*n*=51) | (1) | (50) | (4) | (6) | (18) |
| Axillary node | | | | | |
| metastases | 50% | 100% | 0% | 0% | 0% |
| (*n*=2) | (1) | (2) | (0) | (0) | (0) |
| Matched | | | | | |
| normal | 8% | 44% | 0% | 5.5% | 5.5% |
| (*n*=36) | (3) | (16) | (0) | (2) | (2) |
| Benign | 12.5% | 62.5% | 0% | 0% | 37.5% |
| (*n*=8) | (1) | (5) | (0) | (0) | (3) |

### RESULTS

Amplifiable cDNA, as determined by the ability to amplify β-actin, was obtained from 51 primary breast cancers, 36 samples of matched normal breast tissue (histologically normal tissue taken from the same breast as tumour), two axillary node metastases and eight samples of benign breast tissue.

### Patient details

Clinical and pathological data were available for 49 of the 51 women with breast cancer.

#### *Menopausal status*

Altogether, 77.5% of women were post-menopausal (>6 months since last menstrual period).

#### *Staging*

The majority of tumours were T1 (37%) or T2 (41%), 6% were T3, 12% T4 and 4% of cases were pure in situ disease; 49% of patients were axillary node positive, but none had clinical evidence of distant metastases.

#### *Histology*

Most tumours were invasive ductal carcinomas (86%), 10% were of special histological type (tubular, cribriform, colloid or classic lobular) and 4% were pure ductal carcinoma in situ (DCIS).

#### *Grade*

Over half (55%) of the invasive tumours were poorly differentiated (Bloom and Richardson grade 3), 28% were grade 2 and 17% grade 1.

#### *Oestrogen receptor status (ER)*

Of 39 tumours for which ER status was available, 25 (64%) were ER positive.

 © Cancer Research Campaign 1997

### SSTR mRNA expression

Expression of mRNA for each of the SSTR subtypes was analysed by RT–PCR. Following PCR and resolution on agarose gels, products of amplification were transferred to nylon membranes and hybridized with $\alpha^{32}$P-labelled type-specific cDNA probes. The results of one such analysis for somatostatin receptor subtype 5 expression are shown in Figure 1. Table 2 summarizes the mRNA expression of the five SSTR subtypes in the tissues studied as determined by RT–PCR. SSTR2 was expressed in all but one of the tumours analysed (98%), 44% of matched normal tissue and 62.5% of benign breast tissue. SSTR5 was the next most commonly expressed receptor in tumours (36%). Two of 36 (5.5%) samples of matched normal tissue and three of eight (37.5%) benign tissues expressed this receptor. The remaining three receptor subtypes, SSTR1, 3 and 4, were expressed in less than 13% of cases in all of the tissues analysed. Receptor subtype expression in the two nodal metastases was identical to that of the primary tumours from which they were derived. All but one of the tumours analysed and 50% of matched normal tissues expressed at least one receptor subtype (SSTR2 in virtually all cases). In all, 39% of tumours (all invasive ductal carcinomas) expressed more than one receptor subtype, and tumours of special histological type expressed only SSTR2. Whereas the four tumours that expressed SSTR3 were grade 3, the other receptor subtypes were expressed by all histological grades of tumour and by normal tissues. No tissues expressed all five receptor subtypes. There was no statistically significant correlation between receptor subtype expression and any of the clinical or pathological data mentioned above.

### In situ hybridization

To localize expression of somatostatin receptor subtypes 2 and 5, in situ hybridization was performed on frozen tissue sections. Serial cryostat sections of tumour containing both benign and malignant epithelial cells (as determined by haematoxylin and eosin staining) were selected for hybridization with riboprobes specific for SSTR2 and 5.

#### *SSTR2*

Fourteen tumours and one benign tissue sample were analysed for SSTR2 expression by ISH. Clear cytoplasmic staining was detected in both benign and malignant epithelial cells in all 15 cases. These findings correlated with the results of RT–PCR for all but one of the tumours, which was negative on RT–PCR, but positive on ISH. Matched normal tissue had been analysed by RT–PCR for only three of these 14 tumours and all had been SSTR2 positive. An example of the results of ISH for SSTR2 is shown in Figure 2.

#### *SSTR5*

Ten tumours and one benign tissue section were analysed for SSTR5 expression by ISH. Cytoplasmic staining of normal and malignant epithelial cells was demonstrated in seven tumours, one of which had been negative on RT–PCR. In addition, one tumour that had been positive on RT–PCR was negative on ISH. The section of normal breast tissue was positive on both RT–PCR and ISH.

### DISCUSSION

In this study, we have determined the somatostatin receptor subtype expression in benign and malignant human breast tissue by RT–PCR and ISH. Lack of reliably specific receptor subtype analogues and monoclonal antibodies precludes the detection of receptors at the cell surface and we therefore elected to analyse the steady-state levels of messenger RNA for each receptor subtype. RT–PCR was chosen because it is an exquisitely sensitive technique that will amplify small quantities of messenger RNA, and in many cases only small amounts of tissue were available for analysis. ISH is less sensitive, but allows nucleic acids to be visualized in their cellular environment and was used to localize mRNA expression in the tissues studied.

The most striking observation from this study is that SSTR2 is ubiquitously expressed in breast cancer. Only one tumour out of 51 was SSTR2 negative on RT–PCR, and expression was subsequently detected in this tumour by ISH. This finding is in agreement with previous studies, which have shown that SSTR2 is commonly expressed in human tumours (Eden and Taylor, 1993; Reubi et al, 1994). SSTR2 expression was detected less often in normal breast tissue by RT–PCR (44% matched normal, 62.5% benign/normal), but ISH revealed that histologically normal breast epithelial cells also universally express SSTR2. The apparent lower sensitivity of RT–PCR compared with ISH probably results from the relative paucity of epithelial cells in normal breast tissue compared with tumour. Virtually all the women from whom matched normal tissue was obtained in this study were peri- or post-menopausal (mean age, 60 years; range 42–79 years), an age at which the breast consists primarily of fat with little epithelial tissue identifiable histologically. In fact, matched normal tissue was only available for analysis in 36 of 51 cancer patients because tissue samples from the remaining 15 patients were found to contain no normal epithelial tissue. The benign/normal tissues were obtained from a younger age group (mean age, 45 years; range 26–60 years) undergoing surgery for benign breast disease. In this younger age group, epithelial tissue may have been more abundant, and 62.5% of these tissues were SSTR2 positive. Tissue sections used for ISH were selected for their high content of both benign and malignant epithelium, and the sensitivity of PCR could similarly be improved by microdissection of tissue samples before analysis. A similar pattern of expression was seen for SSTR5, which was the next most commonly expressed mRNA. Approximately one-third of tumours and benign/normal breast tissue expressed this gene, as determined by RT–PCR, compared with only 5.5% of matched normal tissue, suggesting that expression of SSTR5 may be more tumour-specific than SSTR2. However, ISH again demonstrated that when SSTR5 was expressed by tumours, it was also expressed by normal tissue within the same tissue section, suggesting that RT–PCR has underestimated the expression of SSTR5 mRNA in matched normal tissue. The results of ISH indicate that tumours arising in breast tissue expressing SSTR5 mRNA retain this expression, and those arising in SSTR5-negative breast tissue remain SSTR5-negative. SSTR5 is the most recently described of the five SSTRs and, as such, little work has previously been reported on its expression for comparison with these results.

There was good correlation overall between the results for ISH and those for RT–PCR for both SSTR2 and SSTR5. For ISH, all positive cases demonstrated cytoplasmic staining in both normal and malignant epithelial cells, although in some cases staining was heterogeneous. This is most likely to represent artefact, but would be consistent with the results of some affinity binding studies, which have demonstrated heterogeneous distribution of somatostatin receptors (Reubi et al, 1990; Papotti et al, 1989).

© Cancer Research Campaign 1997

The remaining three receptor subtypes (SSTR1, SSTR3 and SSTR4) were expressed infrequently in both tumour and normal tissue, and ISH was not carried out for these receptors. Previous studies have shown that these receptors are less commonly expressed than SSTR2 and are seen mainly in endocrine and gastrointestinal tumours (Eden and Taylor, 1993; Reubi et al, 1994).

The results of this study must be interpreted with caution because the detection of gene expression, as determined by the presence of mRNA, does not necessarily imply the expression of a functional cell-surface receptor. However, it has been shown that the detection of SSTR mRNA by ISH correlates well with cell surface receptor detection by in vitro binding with native somatostatin and its analogues (Reubi et al, 1994).

The data presented in this study are particularly relevant to the application of currently available somatostatin analogues to the treatment of breast cancer, a disease which causes 16 000 deaths per year in the UK. Both direct and indirect growth-inhibitory effects of somatostatin and its analogues have been demonstrated in breast cancer on cell lines in vitro (Setyono-Han et al, 1987; Scambia et al, 1988) and tumour xenografts in vivo (Weber et al, 1989; Noguchi et al, 1993). Indirect growth inhibition is likely to be secondary to a decrease in systemic and local levels of peptides known to be trophic for breast cancer cells, such as growth hormone (Rose et al, 1983) and its mediator insulin growth factor 1 (IGF-1) (Pollack et al, 1989), insulin (Furlanetto and DiCarlo, 1984) and epidermal growth factor (EGF) (Ghirlanda et al, 1983). In this respect, the effects of somatostatin and its analogues have been shown to be synergistic with LHRH agonists (Szende et al, 1989) and tamoxifen (Huynh and Pollack, 1994; Weckbecker et al, 1994). Direct growth-inhibitory effects are mediated via specific high-affinity tumour cell surface receptors, but it is unclear at present which of the five receptor subtypes so far identified are responsible for this effect and which intracellular pathway is involved.

The five SSTRs are G-protein-coupled receptors (GPRs) that exhibit 40–60% overall sequence homology (Yamada et al, 1993). They have a similar affinity for endogenous somatostatin and are all coupled via pertussis toxin-sensitive G-proteins to adenylate cyclase (Patel et al, 1994). However, there are important differences between the receptor subtypes, which suggest that they may mediate different functions of the native peptide. Each receptor subtype has a distinct tissue distribution, different ligand specificities and is linked to different intracellular coupling systems in addition to adenyl cyclase (reviewed in Hofland et al, 1995). Structurally, the greatest homology is between SSTR2, SSTR3 and SSTR5 on the one hand and SSTR1 and SSTR4 on the other, and these structural homologies translate into similar pharmacological profiles (Serrano et al, 1993). The three analogues of somatostatin that are currently in clinical use, octreotide, RC-160 and BIM-23014 (lanreotide), bind with high affinity to SSTR2, SSTR5 and with moderate affinity to SSTR3, but have very low affinity for SSTR1 and SSTR4 (Hoyer et al, 1994).

There is experimental evidence that the direct growth-inhibitory effects of somatostatin are mediated via receptor subtypes 1, 2 and 5. Buscail et al (1995) demonstrated that of the five SSTR subtypes, only SSTR2 and SSTR5 produced growth inhibition in CHO cells, but by different mechanisms, SSTR2 acting via the stimulation of tyrosine phosphatases and SSTR5 via the inhibition of intracellular calcium mobilization. SSTR1 has also been shown to mediate growth inhibition through tyrosine phosphatases, but in different cell lines (COS-7 and NIH 3T3 cells) (Buscail et al, 1994).

In summary, there is a considerable body of experimental evidence to suggest that somatostatin analogues have growth-inhibitory effects in cancer. Indirect growth inhibition is likely to be non-specific, but evidence suggests that direct growth inhibition will be most effective in those tumours that express somatostatin receptor subtypes 1, 2 or 5. In this study, we have demonstrated that two of the SSTRs that mediate direct growth inhibition are expressed in breast cancer. SSTR2 is expressed in all tumours and SSTR5 is expressed in approximately one-third of tumours. Structural analogues of somatostatin with high affinity for both of these receptor subtypes are already available and in safe clinical use, and these analogues may therefore have a significant role in the management of breast cancer.

### ACKNOWLEDGEMENTS

The authors wish to thank Mr RM Rainsbury for allowing his patients to be included in this study and Joanne Waller, Liz Sara and Adrian Bateman for their technical assisstance. This work was supported in part by the people of Guernsey through the Guernsey Research Fellowship administered by the Wessex Medical Trust, which wholly supported SL, and in part by the Cancer Research Campaign.

### NOTE ADDED IN PROOF

Our preferred primers for SSTR3 are now, forward, 5′-ggCCC-TCCCgCCgTgT-3′ and reverse, 5′-CgCTCCTgCCCgCTggT-3′.

### REFERENCES

Buscail L, Delesque N, Esteve J, Saint-Laurent N, Prats H, Clerc P, Robberecht P, Bell G, Liebow C, Schally A, Vaysse N and Susini C (1994) Stimulation of Tyrosine phosphatase and inhibition of cell proliferation by somatostatin analogues: mediation by human somatostatin receptor subtypes SSTR1 and SSTR2. *Proc Natl Acad Sci USA* **91**: 2315–2319

Buscail L, Esteve J, Saint-Laurent N, Bertrand V, Reisine T, O'Carroll A, Bell G, Schally A, Vaysse N and Susini C (1995) Inhibition of cell proliferation by the somatostatin analogue RC-160 is mediated by somatostatin receptor subtypes SSTR2 and SSTR5 through different mechanisms. *Proc Natl Acad Sci USA* **92**: 1580–1584

Chomczynski P and Sacchi N (1987) Single-step method of RNA isolation by acid guanidinium thiocyanate–phenol–chloroform extraction. *Anal Biochem* **162**: 156–159

Eden P and Taylor J (1993) Somatostatin receptor subtype gene expression in human and rodent tumors. *Life Sci* **53**: 85–90

Furlanetto R and Dicarlo J (1984) Somatomedin-C receptors and growth effects in human breast cells maintained in long-term tissue culture. *Cancer Res* **44**: 2122–2128

Ghirlanda G, Uccioli L, Perri F, Altomonte L, Bertoli A, Manna R, Frati L and Greco A (1983) Epidermal growth factor, somatostatin and psoriasis. *Lancet* **1**: 65

Hofland L, Visser-Wisselaar H and Lamberts S (1995) Somatostatin analogs: clinical application in relation to human somatostatin receptor subtypes. *Biochem Pharmacol* **50**: 287–297

Hoyer D, Lubbert H and Bruns C (1994) Molecular pharmacology of somatostatin receptors. *Naunyn-Schmiedeberg's Arch Pharmacol* **350**: 441–453

Huynh H and Pollack M (1994) Enhancement of tamoxifen-induced suppression of insulin-like growth factor 1 gene expression and serum level by a somatostatin analogue. *Biochem Biophys Res Commun* **203**: 253–259

Krenning E, Kwekkeboom D, Reubi J, Van Hagen P, Van Eijck C, Oei H and Lamberts S (1993) $^{111}$In-octreotide scintigraphy in oncology. *Digestion* **54 (Suppl. 1)**: 84–87

Liebow C, Reilly C, Serrano M and Schally A (1989) Somatostatin analogues inhibit growth of pancreatic cancer by stimulating tyrosine phosphatase. *Proc Natl Acad Sci USA* **86**: 2003–2007

 © Cancer Research Campaign 1997

Noguchi S, Nishizawa Y, Motomura K, Inaji H, Imaoka S, Koyama H and Matsumoto K (1993) Inhibitory effect of a somatostatin analogue (SMS 201–995) on the growth of androgen dependent mouse mammary tumor (Shionogi carcinoma). *Jpn J Cancer Res* **84**: 656–663

O'carroll A, Raynor K, Lolait S and Reseine T (1994) Characterisation of cloned human somatostatin receptor SSTR5. *Mol Pharmacol* **46**: 291–298

Papotti M, Macri L, Bussolati G and Reubi J (1989) Correlative study on neuroendocrine differentiation and presence of somatostatin receptors in breast carcinomas. *Int J Cancer* **43**: 365–369

Patel Y, Greenwood M, Warszynska A, Panetta R and Srikant C (1994) All five cloned human somatostatin receptors (hSSTR1–5) are functionally coupled to adenyl cyclase. *Biochem Biophys Res Commun* **198**: 605–612

Pollack M, Polychronakos C and Guyda H (1989) Somatostatin analogue SMS 201–995 reduces serum IGF-1 levels in patients with neoplasmas potentially dependent on IGF-1. *Anticancer Res* **9**: 889–892

Prevost G, Provost P, Salle V, Lanson M and Thomas F (1993) A cross-linking assay allows the detection of receptors for the somatostatin analogue lanreotide in human breast tumours. *Eur J Cancer* **29A**: 1589–1592

Reichlin S (1983*a*) Somatostatin. *N Engl J Med* **309**: 1495–1501

Reichlin S (1983*b*) Somatostatin. *N Engl J Med* **309**: 1536–1563

Reubi J and Torhorst J (1989) The relationship between somatostatin, epidermal growth factor and steroid hormone receptors in breast cancer. *Cancer*, **64**, 1254–1260.

Reubi J, Waser B, Foekens J, Klijn J, Lamberts S and Laissue J (1990) Somatostatin receptor incidence and distribution in breast cancer using receptor autoradiography: relationship to EGF receptors. *Int J Cancer* **46**: 416–420

Reubi J, Schaer J, Waser B and Mengod G (1994) Expression and localisation of somatostatin receptor SSTR1, SSTR2, and SSTR3 messenger RNAs in primary human tumors using *in-situ* hybridisation. *Cancer Res* **54**: 3455–3459

Rohrer L, Raulf F, Bruns C, Buettner R, Hofstaedter F and Schule R (1993) Cloning and characterisation of a fourth human somatostatin receptor. *Proc Natl Acad Sci USA* **90**: 4196–4200

Rose D, Gottardis M and Noonan J (1983) Rat mammary carcinoma regressions during suppression of serum growth hormone and prolactin. *Anticancer Res* **3**: 323-326

Scambia G, Panici P, Baiocchi G, Perrone L, Iacobelli S and Mancuso S (1988) Antiproliferative effects of somatostatin and the somatostatin analog SMS 201–995 on three human breast cancer cell lines. *J Cancer Res Clin Oncol* **114**: 306–308

Schally A, Cai R, Torres-Aleman I, Redding T, Szoke B, Fu D, Hierowski M, Colaluca J and Konturek S (1986) Endocrine, gastrointestinal and antitumor activity of somatostatin analogues. In *Neural and Endocrine Peptides and Receptors*, Moody T (ed.), pp. 73–88. Plenum: New York

Schally A (1988) Oncological applications of somatostatin analogues. *Cancer Res* **48**: 6977–6985

Serrano M, Hannon G and Beach D (1993) A new regulatory motif in cell cycle control causing specific inhibition of cyclin D/CDK4. *Nature* **366**: 704–707

Setyono-Han B, Henkelman M, Foekens J and Klijn J (1987) Direct inhibitory effects of somatostatin (analogues) on the growth of human breast cancer cells. *Cancer Res* **47**: 1566–1570

Szende B, Lapis K, Redding T, Srkalovic G and Schally A (1989) Growth inhibition of MXT mammary carcinoma by enhancing programmed cell death (apoptosis) with analogs of LH-RH and somatostatin. *Breast Cancer Res Treat* **14**: 307–314

Van Eijck C, Krenning E, Bootsma A, Oei H, Van Pel R, Lindemans J, Jeekel J, Reubi J and Lamberts S (1994) Somatostatin-receptor scintigraphy in primary breast cancer. *Lancet* **343**: 640–643

Weber C, Merriam L, Koschitzky T, Karp F, Benson M, Forde K and Logerfo P (1989) Inhibition of growth of human breast carcinomas *in vivo* by somatostatin analog SMS 201–995: treatment of nude mouse xenografts. *Surgery* **106**: 416–422

Weckbecker G, Tolcsvai L, Stoltz B, Pollack M and Bruns C (1994) Somatostatin analogue octreotide enhances the antineoplastic effects of tamoxifen and ovariectomy on 7, 12-dimethylbenz(*a*)anthracene-induced rat mammary carcinomas. *Cancer Res* **54**: 6334–6337

Woltering E, Barrie R, O'dorisio T, Arce D, Ure T, Cramer A, Holmes D, Robertson J and Fassler J (1990) Somatostatin analogues inhibit angiogenesis in the chick chorioallantoic membrane. *Digestion* **46**(suppl. 1): 123

Yamada Y, Post S, Wang K, Tager H, Bell G and Seino S (1992*a*) Cloning and functional characterisation of a family of human and mouse somatostatin receptors expressed in brain, gastrointestinal tract and kidney. *Proc Natl Acad Sci USA* **89**: 251–255

Yamada Y, Reisine T, Law S, Ihara Y, Kubota A, Kagimoto S, Seino M, Bell G and Seino S (1992*b*) Somatostatin receptors, an expanding gene family: cloning and functional characterisation of human SSTR3, a protein coupled to adenyl cyclase. *Mol Endocrinol* **6**: 2136–2142

Yamada Y, Kagimoto S, Kubota A, Yasuda K, Masuda K, Someya Y, Ihara Y, Li Q, Imura H, Seino S and Seino Y (1993) Cloning, functional expression and pharmacological characterisation of a fourth (hSSTR4) and a fifth (hSSTR5) human somatostatin receptor subtype. *Biochem Biophys Res Commun* **195**: 844–852

© Cancer Research Campaign 1997